# Multiple trajectories of alcohol use and the development of alcohol use disorder: Do Swiss men mature-out of problematic alcohol use during emerging adulthood?

**DOI:** 10.1371/journal.pone.0220232

**Published:** 2020-01-27

**Authors:** Mélissa Lemoine, Gerhard Gmel, Simon Foster, Simon Marmet, Joseph Studer

**Affiliations:** 1 Addiction Medicine, Lausanne University Hospital CHUV, Rue du Bugnon, Lausanne, Switzerland; 2 Addiction Switzerland, Lausanne, Switzerland; 3 Centre for Addiction and Mental Health, Toronto, ON, Canada; 4 University of the West of England, Bristol, United Kingdom; 5 Swiss Research Institute for Public Health and Addiction at Zurich University, Konradstrasse, Zurich, Switzerland; Radboud University, NETHERLANDS

## Abstract

**(a) Objective:**

This study aimed to identify trajectories of alcohol use (AU) and their associations with the development of alcohol use disorder (AUD) among young men with different weekly drinking patterns.

**(b) Method:**

A longitudinal latent class analysis integrating several aspects of AU, such as drinking quantity and frequency on weekends vs workweek days, involving 4719 young Swiss men at ages 20, 21, and 25, and collected by the Cohort Study on Substance Use Risk Factors, was used to identify different AU trajectories over time. The development of AUD scores in these trajectories was investigated using generalized linear mixed models.

**(c) Results:**

Six AU trajectory classes, similar to those described in the literature, were identified: ‘abstainers–light drinkers’, ‘light workweek increasers’, ‘light decreasers’, ‘moderate weekend decreasers’, ‘moderate workweek increasers’, and ‘heavy drinkers’. Only 12% of participants were assigned to a trajectory class with decreasing AU associated with a decline in their AUD score. AUD scores increased in trajectory classes exhibiting increasing AU on workweek days, despite low and moderate general AU. Finally, more than 59% of participants were on an AU trajectory presenting no change in their mean AUD score over time.

**(d) Conclusions:**

Maturing out of problematic AU in emerging adulthood is not the norm in Switzerland, and the AUD score developed in late adolescence remains until at least emerging adulthood. AU on workweek days is a more practical marker of potentially problematic AU. This calls for timely interventions in adolescence and concerning regular drinking on workweek days in emerging adulthood.

## Introduction

Developing public social programs requires taking decisions about which populations will be targeted. A target population is generally defined by its life stage, e.g., young adults, or the specific problems it experiences, e.g., problematic alcohol use "[1]".Problematic alcohol use is often more prevalent during emerging adulthood "[2–5]" and drinking seems to increase over time among 18–25-year-olds in most countries "[6]". Heavy drinking, alcohol problems, and alcohol use disorders (AUD) generally peak as people go through late adolescence and then decline as they grow older "[7, 8]". This process of *maturing out* of problematic alcohol use (based on "[9]") seems to coincide with the adoption of social roles (e.g., parenthood, employment, conjugal relationships "[10, 11]").

However, problematic alcohol use can take different forms "[12]". Studies have generally used heavy episodic drinking (HED), the consumption of a large quantity of alcohol on a single occasion, to quantify alcohol use (AU) in adolescence because it is the dominant AU pattern during this specific period "[5]". Moreover, HED is commonly associated with high-volume drinking, and heavy episodic drinkers are more likely to experience alcohol-related consequences such as AUD, violence, accidents, and poisoning (e.g., "[13–15]"). Alcohol consumption can be described using additional measurements, such as frequency of use, typical quantity, or average drinking volume (DV). Each measurement captures different aspects of individuals’ consumption patterns "[14, 16–18]". Indeed, multiple-trajectory approaches based on separate alcohol indices tend to class individuals differently (e.g., "[16, 18]" despite strong correlations between the indices "[14, 19]").

In addition, adolescents can present very contrasting drinking patterns over the week: some gradually increase their use from Monday to Sunday, whereas others drink mostly at weekends, mainly on Friday and Saturday nights "[20, 21]". Integrating the multidimensionality of AU using a weekly timescale may therefore be particularly relevant when quantifying the heterogeneity of AU in young adults. Drinking at weekends may be associated with leisure and party activities with friends—young adults drinking to seek excitement and fun "[22]". Conversely, drinking on workweek days may be related to the stresses of employment, such as lack of job security, work fatigue, interpersonal conflict, or poor leadership "[23]". In such situations, alcohol may be used as self-medication "[24]", and the motivations for drinking would therefore be different. Enhancement motives (*drinking to get high*) are generally more strongly associated with AU at weekends, whereas coping motives (*drinking to manage unpleasant feelings*) are more strongly associated with AU on workweek days "[25, 26]". This distinction could be important since drinking to self-medicate anxiety may be associated with the development and the persistence of AUD "[27]". Consequently, while drinking at weekends and on workweek days may be interconnected, they could result from different processes and lead to different problematic alcohol use over time.

One way to better characterize and understand the multiple forms of AU is to examine the developmental trajectories of AU over time using a multiple-trajectory approach "[12, 28]". The present study used this approach to explore the multiple forms of AU varying in time among young men ageing from 20 to 25 years old and to identify the trajectories of AU reflecting the development of different patterns of drinking. To characterize these drinking patterns, we used some commonly used AU aspects, such as quantity, frequency and HED frequency, and specifically investigated different patterns of drinking over the week (i.e., weekend vs workweek).

Previous studies describing AU trajectories generally found four to five trajectories during the transition from late adolescence to young adulthood (reviewed in "[29]"). A low or no drinking trajectory, i.e., when alcohol consumption remains low throughout time, was the most common pattern in the populations studied, followed by a decreasing (or inverse U-shaped) trajectory involving adolescents drinking heavily but maturing out of that drinking behavior. Finally, two additional though less prevalent trajectories are generally described, associated with the greatest risk of AU problems: a group with rapidly escalating AU, and a group with chronically high-drinking volumes "[29]". When studying the transition from late adolescence to young adulthood, some studies identified a peak of AU in the early 20s (e.g., "[14, 18]"), whereas others suggested a peak around or after the mid-20s (e.g., "[29–31]"). However, research on AU trajectories mainly focused on English-speaking countries, which differ in drinking style from grape-growing, wine-drinking south-western European countries like France, Switzerland, Spain, and Italy "[6, 32]", where the shapes and prevalence rates of AU trajectories are, to the best of our knowledge, undocumented. We therefore expected to reveal at least four trajectories but made no predictions as to their shape or prevalence. In term of AU measures, we expected HED frequency to decrease over time. However, for heavy episodic drinkers, the specific pattern of HED can be replaced by another drinking pattern, as problematic as HED. Indeed, the increase of drinking during workweek days or a heavy drinking during week-end can be more likely in heavy episodic drinkers.

Furthermore we evaluated whether these drinking patterns can translate into AUD, a problematic AU with clinically significant impairment or distress. The AU trajectories may predict the risk of developing an AUD in adulthood: early heavy drinkers or fast increasers were more likely to be diagnosed with an AUD at a later stage "[33, 34]". Trajectories on alcohol symptomology generally identify between three to six trajectories, very similar to AU trajectories "[31, 35–37]". Moreover AUD trajectories can be predicted by previous HED "[37]". Despite the association between HED and drinking volume, they can have independent and combined effects on AUD "[38]". Although it seems intuitive that AUD is linked to heavy AU "[39]", few studies have investigated the association between the development of AUD and the changes of AU (e.g., "[40, 41]"). Chung et al. (2005) found that symptom severity was moderately related to AU patterns over one year while Dawson et al. (2008) showed that changes in consumption were associated with AUD transition over three years. Consequently we predicted that, over five years, AUD scores would follow the AU trajectories. Specifically, we investigated whether participants’ AUD scores were higher and increased faster among heavy drinkers and faster increasers, than among participants with other AU trajectories.

## Materials and methods

### Study design and participants

Participants were enrolled in the Cohort Study on Substance Use Risk Factors (C-SURF), a longitudinal study designed to assess substance use patterns and risk factors among young Swiss men. Enrolment took place in three of Switzerland’s six army recruitment centers, located in Lausanne (French-speaking), Windisch, and Mels (German-speaking), covering 21 of the country’s 26 cantons. Army recruitment procedures are mandatory for all young Swiss men around 19 years old; there is no preselection for conscription. The sample can consequently be considered to be representative for young Swiss men in general, covering most cantons and reflective of both main languages, and rural and urban regions. Army recruitment was used to inform and enroll participants, but study assessments were carried out outside of the army environment, independent of its influence and individuals’ eligibility for military service. The study protocol was approved by the Clinical Research Ethics Committee of Lausanne University (Protocol No. 15/07). We obtained a written consent of the participants and the data are analyzed anonymously.

### Measures

#### Alcohol use at weekends

Quantity and frequency of AU on weekend days (Friday to Sunday) were assessed for the last 12 months. Frequency was measured by the usual number of days on which participants drank at least one standard drink, on a 7-point scale ranging from ‘never’ to ‘every weekend day’. Quantity was measured by the average number of standard drinks consumed on use days, on a 6-point scale (‘one or two’, ‘three or four’, ‘five or six’, ‘seven or eight’, ‘nine to eleven’, ‘twelve or more’), but was analyzed including ‘none’ for participants who had not drunk during the last 12 months. Standard drinks containing approximately 10–12 g of pure alcohol were illustrated graphically.

#### Alcohol use on workweek days

Quantity and frequency of AU on workweek days were assessed from Monday to Thursday. Frequency was measured on an 8-point scale ranging from ‘never’ to on ‘every workweek day’ and was analyzed using the same scale as frequency on weekend days (by creating an item category ‘at least three days per week’) to ensure a sufficient number of participants in each category for the statistical analysis. Quantity on workweek days was measured as for weekend days and analyzed on a 4-point scale by collapsing the four last item categories into ‘five drinks or more per day’.

For descriptive purposes, the drinking volume (DV), measured by the average number of drinks per week over the previous twelve months, was calculated using a quantity–frequency approach. Total DV per week was estimated by summing DV on weekend and workweek days. A heavy drinking volume was defined as more than 21 standard drinks per week "[42]".

#### Heavy episodic drinking (HED)

Participants were asked how often they drank a quantity of six standard drinks of 10-12g of pure alcohol (which corresponds to five standard drinks containing 14g of pure alcohol) or more on a single occasion over the previous twelve months. Answers were collected on a 5-point scale (‘never’, ‘less than once a month’, ‘every month’, ‘every week’, ‘nearly every day’), but the item categories ‘every week’ and ‘nearly every day’ were collapsed to ensure a sufficient number of participants in each item category for the statistical analysis. The prevalence of monthly HED, i.e., participants drinking six drinks or more on a single occasion at least monthly, was calculated for descriptive purposes "[43]".

#### Alcohol use disorder (AUD)

AUD scores were based on the eleven criteria defined in the DSM-5, as experienced in the previous 12 months "[44, 45]". Criteria were summed to get AUD scores ranging from 0–11.

### Statistical analyses

#### Longitudinal latent class analysis (LLCA)

To characterize participants’ AU over time, a mixture of multivariate multinomial distributions was used to create latent classes with similar patterns of drinking frequency and quantity on workweek and weekend days and HED frequency over time: these are hereafter referred to as AU trajectories. LLCA allows the identification of AU trajectories based on multiple non-Gaussian response variables at three time-points with different time intervals "[46–48]". However, by providing latent class membership probabilities for each individual, and item-response probabilities conditional on latent class membership "[47]", latent class analyses ‘only’ allow qualitative estimations of trends over time. Although latent growth mixture modeling would be preferable for describing quantitatively latent trajectories, latent growth mixture models require making assumptions which could substantively affect their interpretation "[49]". Importantly, when time intervals vary, non-linear trajectories would be preferable, but they are generally restricted to functions requiring more than three points in time, such as polynomial or piecewise models. In contrast, by assuming local independence ‘only’ "[46, 47]", LLCAs estimate a large number of parameters without constraining the distribution of the observed variables or the form of change "[48]".

The LLCA was performed using the MixtComp library available on the Massive Clustering with Cloud Computing platform (https://massiccc.lille.inria.fr/help/libraries). The presence of 1–20 classes was investigated as follows. For each number of classes, model estimation was replicated 20 times to avoid local maxima solutions. Then, the model with the best Bayesian information criterion (BIC) was chosen and used in the comparison to determine the optimal number of latent classes. The optimal number of latent classes was determined by the BIC, the relative entropy, and the average posterior probability by class. The BIC is an indicator of the trade-off between the model’s goodness-of-fit to the data and its complexity. The LLCA’s BIC was calculated according to "[50]" and is always negative: thus, the best BIC is the one closest to zero. Relative entropy approaching 1 indicates clear delineation of classes "[51]"; an average posterior probability by class greater than 0.9 indicates high classification quality "[28, 52, 53]" advised basing model selection on entropy when models have similar BIC. The interpretation of the classes derived was based on the heat map of item-response probabilities (see “S1 Table” for the values).

#### Predicting mean levels of AUD over time

After selecting the optimal number of classes, mixed models including the most likely class membership as the between-subject factor, time as the within-subject factor, and the interaction between class membership and time were used to predict the AUD score (equivalent to a repeated measures ANOVA for non-Gaussian variables). Longitudinal trajectories were modelled with a random intercept per participant, to account for the non-independence of data from the same participant, and with time as a fixed categorical variable, to estimate the non-linear effect of time over the cohort study’s three response periods, or waves.

Since the sample included abstainers and heavy drinkers, a count variable such as the AUD score can be differently over-dispersed over AU trajectories. The AUD score was therefore modelled using a generalized linear mixed model (GLMM) consisting of a mixture between a negative binomial distribution and a structural component to model dispersion according to the additive effect of AU trajectories and time. We used the R software package ‘glmmTMB’ "[54]". This model outcompeted other models, such as a model based on a zero-inflated Poisson distribution (see “S1 Supplementary Material” and “S2 Table”). The statistical significance of the interaction between time and AU trajectories was assessed using a likelihood ratio test (LRT). *Post hoc* Wald tests based on z-statistic values were done to determine which between-group differences were different from zero. To evaluate the robustness of the results, a generalized estimating-equations model was also run (see “S1 Supplementary Material”). Results are compared in “S3 and S4 Tables”, and they differed only for two ‘marginal’ effects (*P* > 0.04) that were therefore not reported in the result section.

#### Sample size and missing data

A total of 5987 participants filled in the baseline questionnaire between September 2010 and March 2012. Among them, 4794 (80.1%) completed the two follow-up questionnaires (March 2012–January 2014 and March 2016–July 2017). An average of 15.79 ± 3.33 months and 49.42 ± 4.59 months separated the first and second assessments, and the second and third assessments, respectively. Due to the exploratory and descriptive purpose of the study, aiming to explore the heterogeneity of AU over time and to identify different trajectories based on correlations among indices, listwise deletion was favored rather than missing value imputations that often require predictive models with assumptions on the distribution of the variables and their covariance. At the end, three points by variable by individual, which are required to robustly estimate variance and temporal trends, were included in the statistical analysis. Overall 4746 participants (99.0% of respondents who answered three questionnaires) were included in the AU trajectory analysis and 4719 participants (98.4% of respondents who answered three questionnaires) in the AUD analysis. A previous study reported a small non-response bias between respondents and non-respondents at wave one "[55]".

The questionnaires can be found on the website of C-SURF (https://www.c-surf.ch/fr/2.html) and the data analyzed on the website of Zenodo (10.5281/zenodo.2636737).

## Results

The average ages of participants in response waves 1, 2, and 3 were 19.97, 21.28, and 25.40 years old, respectively. Out of 4746 participants, 56.70% were French-speaking. Descriptive statistics of all alcohol-related variables over the three waves are summarized in “Table 1”.

**Table 1:** Description of AU among participants over the 3 waves.

Variable	Wave 1	Wave 2	Wave 3
Age	19.97 ± 1.23	21.28 ± 1.26	25.40 ± 1.24
DV weekend	6.88 ± 7.98	6.51 ± 7.44	5.52 ± 6.74
DV workweek	1.18 ± 3.10	1.42 ± 3.03	1.64 ± 3.66
Total DV	8.06 ± 9.80	7.93 ± 9.28	7.16 ± 9.27
% DV workweek	12.1	15.7	20.4
% Heavy vol. drinkers	9.9	9.0	7.3
% Monthly HED	50.0	43.9	38.0
AUD score	1.24 ± 1.71	1.22 ± 1.61	1.24 ± 1.60
% AUD (> 2 criteria)	31.3	31.8	32.0
% Severe AUD	3.0	2.5	2.2

Observed means ± standard deviation; DV: drinking volume (number of standard drinks per week); % DV workweek days: average percentage of DV drunk on workweek days in comparison to the total DV; % Heavy volume drinkers: prevalence of participants drinking more than 21 standard drinks per week; % monthly HED: prevalence of participants drinking at least six drinks on a single occasion, once monthly or more; AUD: alcohol use disorder; % AUD: prevalence of participants scoring at least 2 on the AUD scale; % severe AUD: prevalence of participants scoring at least 6 on the AUD scale (severe AUD).

### Optimal number of latent classes

**Fig 1:**
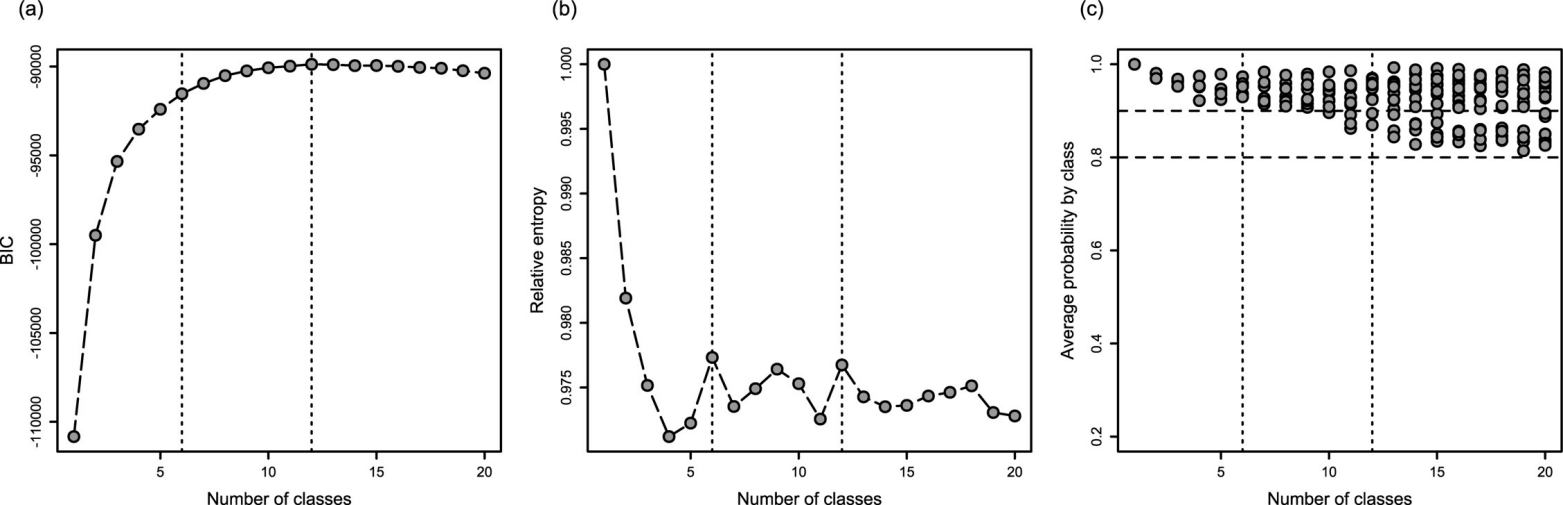
Performance of the LLCA models for 1 to 20 latent classes: (a) the BIC, (b) the relative entropy, and (c) the average posterior probability by class. The dotted lines highlight the results for 6 and 12 latent classes. The dashed lines indicated the average probability by class thresholds of 0.8 and 0.9.

BIC, relative entropy, and average posterior probabilities for the 1–20 LLCA classes are shown in “Fig 1”. BIC provided support for a solution involving at least 4 classes. Among solutions with 4 classes or more, relative entropy and average posterior probabilities provided support for the 6-class and 12-class solutions. The 6-class solution, with the best relative entropy, was retained for its ease of interpretation. The relative entropy (> 0.970) and high average probability by class (> 0.9), for all classes, also suggested that the classification was good and that so was the model’s discrimination.

### AU trajectories based on LLCA

**Fig 2:**
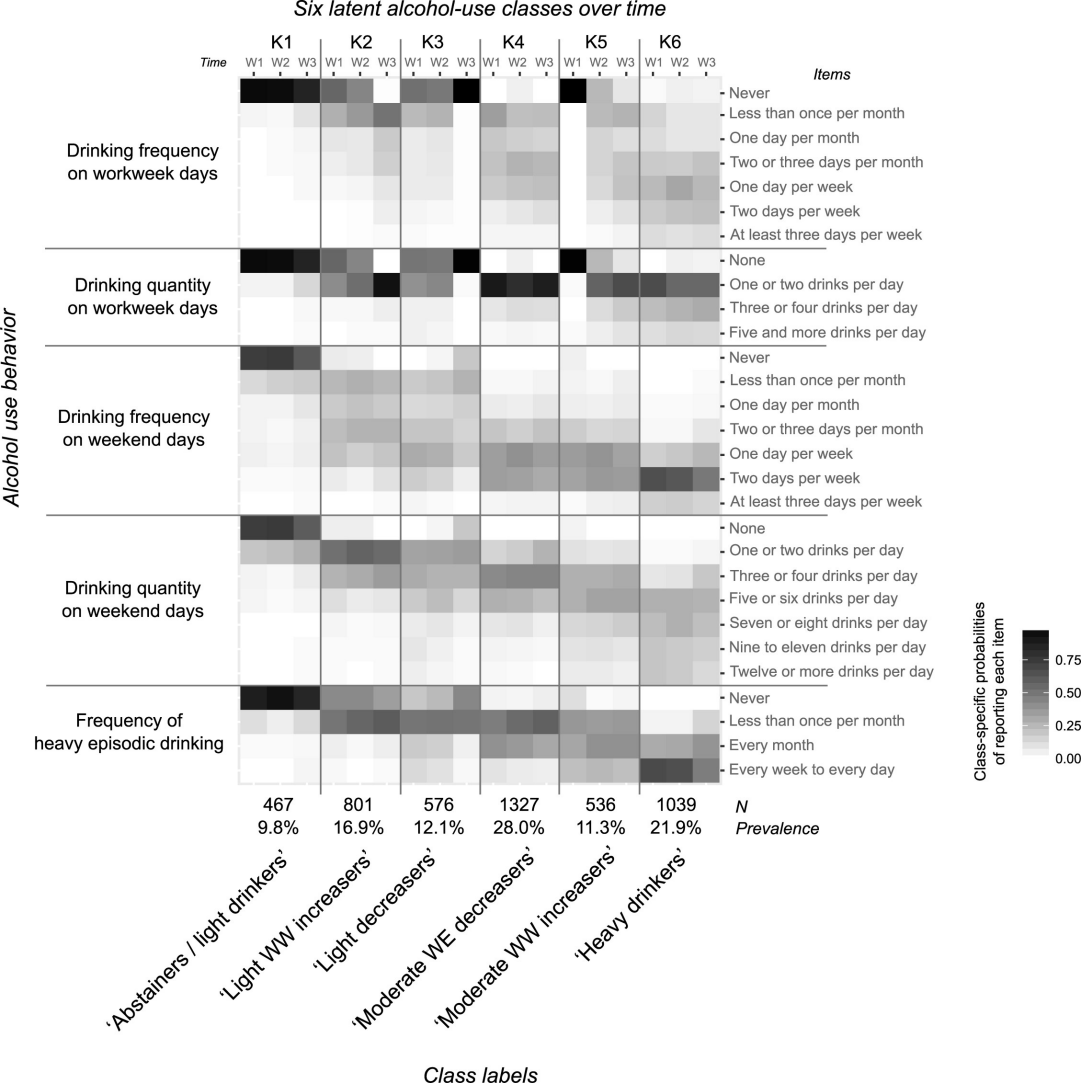
Heatmap of the characteristics of the six AU trajectories based on the class-specific probabilities of reporting each AU behavior (i.e., item).

Characterization of the 6 AU trajectories was based on the class-specific probabilities of reporting each AU behavior from the LLCA (“Fig 2”, “S1 Table” for values). “Table 2” summarizes the AU trajectories by DV, HED, and AUD prevalence, with 9.8% of young Swiss men being mainly characterized by abstinence on workweek days, very low AU on weekend days, and rare HED (“Fig 2”: K1 –*abstainers–light drinkers*). A second trajectory of low-users at age 20 (16.9%) was characterized by a clear increase in their AU on workweek days and a relatively stable (or slightly increasing) pattern of moderate AU on weekend days and HED over time (“Fig 2”: K2 –*light workweek (WW) increasers*). In contrast, 12.1% of participants were on a trajectory that had matured out by age 25 (“Fig 2”: K3 –*light decreasers*), switching from drinking to not drinking (‘never’ and ‘none’) on workweek days. In addition, the probability of not drinking (answering ‘never’ or ‘none’) increased over time for drinking frequency and quantity on weekend days, as it did for the frequency of HED. Two trajectories of moderate drinkers were also found. Some were assigned to a trajectory (28.0%, “Fig 2”: K4 –*moderate weekend (WE) decreasers*) that decreased its frequency of HED and drinking quantity on weekends and simultaneously kept its moderate drinking quantity on workweek days (i.e., mainly drank ‘one or two drinks per day’ between 20 and 25 years old). The other trajectory of moderate drinkers (11.3%, “Fig 2”: K5 –*moderate WW increasers*) was characterized by a clear increase in AU on workweek days since its participants switched mainly from ‘never’ drinking to drinking at different frequencies, but mostly less than three days per week on workweek days. This behavior was combined with an increased frequency of HED. Finally, 21.9% of participants were assigned to a trajectory of stable heavy drinking, whose alcohol use on weekends and frequency of HED slightly decreased over time but stayed relatively high (“Fig 2”: K6 –*heavy drinkers*).

**Table 2:** Description of alcohol use in terms of observed average drinking volume (on workweek days, at weekends, and total DV) and AUD prevalence according to its severity over the 3 waves by alcohol use class.

AU classes	K1			K2			K3			K4			K5			K6		
Labels	Abstainers–			Light WW increasers			Light decreasers			Moderate WE decreasers			Moderate WW increasers			Heavy drinkers		
	light drinkers																	
N_AU_ (%)	467 (9.8%)			801 (16.9%)			576 (12.1%)			1327 (28.0%)			536 (11.3%)			1039 (21.9%)		
Wave	W1	W2	W3	W1	W2	W3	W1	W2	W3	W1	W2	W3	W1	W2	W3	W1	W2	W3
DV workweek	0.06	0.04	0.14	0.23	0.25	0.74	0.71	0.59	0.02	1.12	1.32	1.46	0.00	1.29	2.08	3.38	3.58	3.93
DV weekend	0.30	0.15	0.74	1.84	1.18	2.02	4.94	3.75	1.76	5.69	5.39	4.55	7.06	8.39	7.67	16.22	15.47	12.57
Total DV	0.36	0.19	0.88	2.07	1.44	2.76	5.65	4.34	1.78	6.80	6.71	6.00	7.06	9.67	9.76	19.59	19.05	16.50
% DV workweek	17.0	22.4	15.9	11.0	17.7	26.9	12.5	13.6	0.9	16.4	19.7	24.2	0.0	13.3	21.4	17.2	18.8	23.8
% monthly HED	1.7	0.6	4.1	8.6	2.5	6.5	30.9	25.5	7.1	48.6	42.6	35.9	52.8	66.7	62.1	96.2	95.6	85.1
N*		466			797			575			1319			533			1028	
AUD prev.—No	97.9	98.3	97.0	87.8	88.2	84.2	79.8	80.5	88.0	67.6	67.7	66.9	71.5	60.8	58.4	34.5	36.7	37.3
AUD prev.—Mild	1.5	1.1	2.8	10.0	10.4	12.8	13.7	14.1	10.1	24.7	27.2	27.5	20.5	28.9	30.6	40.3	38.0	38.8
AUD prev.—Mod.	0.2	0.4	0.2	1.8	1.3	2.1	3.1	4.4	1.2	5.9	4.2	4.7	5.4	6.4	8.8	17.3	17.7	17.5
AUD prev.—Sev.	0.4	0.2	0.0	0.4	0.1	0.9	3.3	1.0	0.7	1.8	0.9	1.0	2.6	3.9	2.3	7.9	7.6	6.4

DV: drinking volume (number of standard drinks per week). % monthly HED: prevalence of participants drinking at least six drinks on a single occasion, once monthly or more. Prevalence of an alcohol use disorder according to its severity: No (0–1 criterion), Mild (2–3 criteria), Mod.: moderate (4–5) and Sev.: severe (6 or more).

* Due to missing values, 4746 participants were included in the analysis on AU trajectories, whereas 4719 participants were included in the analysis on AUD.

Overall, the frequency of HED and drinking quantity on weekend days increased gradually over trajectories, from *abstainers–light drinkers* to *heavy drinkers* (“Fig 2”, “S1 Table”). This increase was less gradual for drinking frequency on weekend days: differences between *light WW increasers* (K2) and *light decreasers* (K3), and between *moderate WE decreasers* (K4) and *moderate WW increasers* (K5), were not apparent (“Fig 2”, “S1 Table”). In comparison, drinking on workweek days contrasted between trajectories (“Fig 2”, “S1 Table”), for example: those who started to drink on workweek days (*moderate WW increasers* (K5)) vs. those who stopped (*light decreasers* (K3)); those who increased drinking quantity (*light WW increasers* (K2)) vs. those exhibiting stable moderate use (*moderate WE decreasers* (K4)). In others words, characteristic behaviors within trajectories (> 80% of the participants within trajectories, “S1 Table”) were identified for quantity and frequency on workweek days and for HED frequency. For example, a characteristic behavior for quantity on workweek days was found in 5 out of 6 trajectories. None was observed for quantity and frequency on weekend days (apart from the absence of some items answering mainly ‘never’ and ‘none’). Overall, the trajectories described a gradient for AU at weekends where the boundaries between trajectories appeared fuzzy, whereas the characterization of the trajectories, based on AU on workweek days, was categorical over time: those who stopped, those who started, those who were stable, and those who increased.

### Mean levels of AUD within AU trajectories

AUD scores varied across the six AU trajectories, both in terms of their initial levels and temporal variation (trajectory by time interaction: LRT *χ*^2^_10_ = 104.00, *P* < 0.001; “Table 3”). Based on pairwise comparisons, the six AU trajectories exhibited five different levels of AUD score at wave 1 (“Table 3”, and “S3 Table” for detailed information). *Heavy drinkers* (K6) had more AUD criteria than moderate drinkers (K5 and K4), who had more criteria than *light decreasers* (K3), who had more than *light increasers* (K2), who had more than *abstainers–light users* (K1). In contrast, at wave 3, the six AU trajectories exhibited six different levels of AUD score (“Table 3”, and “S3 Table”). *Heavy drinkers* (K6) had more AUD criteria than *moderate WW increasers* (K5), who had more than *moderate WE decreasers* (K4), who had more than *light WW increasers* (K2), who had more than *light decreasers* (K3), who had more than *abstainers–light drinkers* (K1). Over time, the number of AUD criteria significantly decreased for *light decreasers* (K3), increased for *light WW increasers* (K2) and *moderate WW increasers* (K5), and did not vary for *abstainers–light users* (K1), *moderate WE decreasers* (K4), and *heavy drinkers* (K6) (“Table 3”, and “S4 Table”).

**Table 3:** Mean alcohol use disorder (AUD) score over time by AU trajectory.

AU trajectory	Mean AUD score over time			
Labels	W1	W3	Δ_W2-W1_	Δ_W3-W2_
K1—Abstainers–light drinkers	0.09^a^	0.13^a^	0.00^NS^	0.04^NS^
K2—Light WW increasers	0.51^b^	0.67^c^	-0.05^NS^	0.21***
K3—Light decreasers	0.95^c^	0.55^b^	-0.15*	-0.25***
K4—Moderate WE decreasers	1.26^d^	1.22^d^	-0.09^NS^	0.05^NS^
K5 –Moderate WW increasers	1.15^d^	1.55^e^	0.32***	0.08^NS^
K6—Heavy drinkers	2.50^e^	2.42^f^	-0.03^NS^	-0.05^NS^

Mean AUD score over time: W1 and W3: observed means by class (score) at waves 1 and 3 respectively. Lower case letters (^a^, ^b^, etc.) indicate independent contrasts significant at *P* < 0.05 based on *post hoc* Wald tests. Δ_W2-W1_ and Δ_W3-W2_: observed differences by class between wave 2–wave 1 and between wave 3–wave 2, respectively, and their significance based on *post hoc* Wald tests.

NS: non-significant

* *P* < 0.05

** *P* < 0.01

*** *P* < 0.001.

Predicted values are given in “S3 and S4 Tables”.

## Discussion

Based on the frequency of HED and on drinking frequency and quantity on workweek and weekend days over three waves of questionnaire responses, the present study sought to identify AU trajectories in young Swiss men. By explicitly discriminating between drinking on weekend and workweek days, we aimed to quantify the heterogeneity of AU based on contrasting drinking patterns that probably reflect different social, environmental, and motivational drinking contexts.

### In general, alcohol use decreases as Swiss men move from 20 to 25 years of age

Overall, six AU trajectory classes were identified: *abstainers–light drinkers* (9.8%), *light WW increasers* (16.9%), *light decreasers* (12.1%), *moderate WE decreasers* (28.0%), *moderate WW increasers* (11.3%), and *heavy drinkers* (21.9%). This classification was very similar to the AU trajectories reported in previous studies: low/no drinking class, small escalating class, developmentally limited class, late-onset class, and a chronically high-level class (for review, see “[29]”). Overall, general AU, and use on weekend days especially, decreased over time: *light decreasers* (K3), *moderate WE decreasers* (K4), and *heavy drinkers* (K6), representing 62.0% of the total population sampled, showed decreasing AU over time. This observation was congruent with evidence from longitudinal studies in English-speaking countries, which showed that AU peaked at late adolescence and subsequently declined as people grew older "[14, 18, 29, 30]".

However, in contrast to the general decrease in AU on weekend days, AU on workweek days showed clear, distinctive trends in certain trajectory groups, which did not support the idea of a maturing-out process. Specifically, AU on workweek days clearly increased over time in two trajectory classes (the *light WW increasers*, K2, and *moderate WW increasers*, K5).

### Do young Swiss men mature out of problematic AU?

In parallel to the measurement of AU over time, the AUD score—a measure of alcohol-related problems—was also monitored over time within the AU trajectory classes to identify variations in AU that might result in changes in alcohol-related problems. The mean level of AUD increased gradually across the AU trajectories classes (from K1 to K6), as did the mean level of AU. More than 59% of participants were in AU trajectory groups (i.e., *abstainers–light drinkers*, *moderate WE decreasers*, *heavy drinkers*) which presented no significant increases or decreases in mean levels of AUD over time. Of the three trajectory classes where AU decreased, the mean level of AUD only decreased among the *light decreasers*, where men not only reduced their frequency of HED and AU on weekends but also reduced drinking on workweek days. More precisely, there was no evidence of a decrease in mean AUD scores over time in trajectory classes where AU on workweek days was stable or slightly increased despite observed declines in the frequency of HED and AU on weekends (*moderate WE decreasers*, K4, and *heavy drinkers*, K6). These results suggested that only *light decreasers* reduced their AU sufficiently to also reduce their AUD score, and therefore matured out of their potentially *problematic* AU.

Overall, our results suggested that a change in AU on workweek days could be a more practical indicator of the risk of developing an AUD than a change on weekends. Firstly, a change in the level of AUD followed a change in AU on workweek days rather than on weekend days. Indeed, mean levels of AUD only declined in the trajectory class showing a decrease in AU on workweek days, and it increased in the two trajectory classes—*light increasers* and *moderate WW increasers*—that increased their AU over time on workweek days, when no clear pattern was found for weekends. Second, the characterization of the trajectories, based on AU on workweek days, was mainly categorical over time: those who stopped, those who started, those who were stable, and those who increased, whereas the boundaries between trajectories appeared fuzzy for AU on weekends.

Alcohol addiction, diagnosed via AUD, can be seen as “an aberrant form of learning, where alcohol exposure leads in time to alteration in the neurocircuitry underlying stress response, reward and cognitive functioning, all of which ultimately leads to compulsive substance use” (reviewed in "[56]"). Therefore both regularity and stress can play a role in the development of addictions. Alcohol use on workweek days could indicate a more regular drinking pattern than drinking only during week-ends, and therefore a behavior more likely to progress to addiction. Alternatively, alcohol use on workweek days could reflect self-medication and coping motives, suggesting high perceived stress or a mindset where stress is perceived as negative (and not stimulating). Both can lead to higher stress hormones (e.g., cortisol) known to influence the brain’s reward system as well as cognitive processes, and may contribute to alcohol’s reinforcing effects, habit formation and risk of relapse "[57]". This may explain why individuals can be diagnosed with an AUD without heavy AU, and inversely why individuals can drink heavily without being diagnosed over time with an AUD "[58, 59]".

### Potential reasons for not maturing-out

Overall, 12% of young Swiss men were in a trajectory class showing a decline in both AU and AUD score over time, whereas 50% were classed as moderate drinkers and heavy drinkers who slightly decreased their AU while their AUD score did not change over time. Maturing out of problematic alcohol use between 20 and 25 years old was therefore not a normative development in young Swiss men. One possible reason for this is that moderate and heavy drinkers could only *start* the process of maturing-out, whereas *light decreasers* were in the stage of *finishing* that process. Indeed, when studying the transition from late adolescence to young adulthood, some studies have identified a peak in AU in the early 20s, leading to a general decline over time of both alcohol consumption and alcohol-related problems (e.g., "[14, 18]"). However, other studies also suggested a peak around or after the mid-20s (e.g., "[29–31]"). Today, the adoption of social roles that have traditionally defined adulthood (parenthood, employment, conjugal relationships) are postponed "[60, 61]". Recent studies suggested that this pattern occurs also in Switzerland "[62, 63]". Indeed, Kuntsche et al. (2016) found that an early engagement in permanent social roles is uncommon in Switzerland. And C-SURF participants endorsed on average less than two of the five common social roles (i.e., completed education, living independently from parents, financial independence, stable relationship and parenthood) at age 25 "[63]". Life course trajectories in young adults also appear to have become less standardized and more individualized "[60, 64]". More relaxed social norms have resulted in a wider range of lifestyle choices "[1]". Thus, Swiss men born in the early 1990s may have delayed their process of maturing out of problematic AU.

Alternatively, the process of maturing-out may be less important in Switzerland: as a wine-growing country with the stereotypical drinking pattern of a *wet drinking* culture where AU is often less extreme but a more regular everyday act throughout a person’s life "[32]". Indeed, in Switzerland "[65]" and France "[66]", total DV was found to peak after 60 years of age, whereas HED seemed to decrease from late adolescence into emerging adulthood. However, these two national studies were based on cross-sectional data and should therefore be interpreted with caution, since longitudinal and cross-sectional studies are not directly comparable. Differences were also found between regions in Switzerland where drinking patterns follow a *dry drinking* culture in the German-speaking part with beer as a favorite beverage and a *wet drinking* culture, around wine, in the French-speaking part "[67]". However, this different enculturation commonly happens after 25 years of age "[68]". Consistently, no difference in prevalence of LLCA classes was found between the French and German speaking parts of Switzerland (data not shown). However the absence of difference in prevalence between French and German speaking parts of Switzerland does not rule out the existence of subtle cultural differences between linguistic and cantonal regions but will require further in-depth analyses including for example the cantonal legislation on drinking "[69, 70]" as well as outlet density "[71]".

### Limitations

The present study’s strengths included a large sample and robust longitudinal records of alcohol use on both workweek and weekend days. However this study had some limitations. First the definition of working week was based on standard office hours and was the same for all individuals. Therefore the working time distribution may not capture the individual variation or the working time of some specific categories such as catering, cleaning or security personnel and shift work. *Drinking during workweek days* could therefore result from specific drinking motives such as coping with work related stress but also from drinking outside of working days for working categories with different working time distribution than used in this study. An individual diary reporting working time and alcohol use would be necessary to fully explore the link between working time and problematic alcohol use.

Second, LLCA performed on multiple non-Gaussian response variables qualitative estimations of trends over time. The inclusion of more waves would allow to describe quantitatively the latent trajectories using latent growth mixture modeling in order to confirm the temporal trends and the correlations among AU measures as well as to discriminate between individuals who mature-out late in life and individuals who decrease their AU without maturing out of alcohol-related problems (e.g., AUD). Moreover in the analysis, only participants with at least three time-points were included while missing data were handled using listwise deletion, which can bias the estimates and affect the representability of the sample.

Lastly, the sample was composed solely of Swiss men and five cantons were missing, therefore the results cannot be generalized to the entire Swiss population. Although the development of drinking behavior is similar between men and women "[72]", men generally report higher levels of drinking, heavy drinking and AUD prevalences "[72, 73]". In term of AU trajectories, evidences are inconsistent with some studies finding gender differences (e.g., "[33]") and others not so much "[18]". Therefore trajectories of alcohol use and the process of maturing-out of problematic alcohol use need to be investigated also in Swiss women.

## Conclusions

The development of AU as young Swiss men age from 20 to 25 is not homogenous. The present study identified six different trajectories in line with previous studies investigating AU trajectories. Only 12% of the participants were assigned to a trajectory class exhibiting the decreasing AU associated with a decline in mean AUD score. This suggested that maturing out of alcohol use in emerging adulthood was not the norm in Switzerland. Moreover, more than 59% of participants were assigned to an AU trajectory class which presented no variation in its AUD score over time. This result suggested that an AUD developed in late adolescence will remain until at least emerging adulthood. This points to the importance of focusing interventions on preventing the development of AUD early on in life—before the age of 20. Nevertheless, our results supported the idea that people who rapidly increase their AU are at the greatest risk of developing an AUD, despite their low initial level of consumption "[29]". Moreover, a change in AU on workweek days was a good marker of the development of an AUD, although AU on workweek days only represents a small fraction of total DV. Thus, research should aim to understand the motivations behind drinking on workweek days, and prevention programs should target them accordingly.

## Supporting information

S1 Supplementary Material Supplementary methods on the GMM and GEE. (DOCX)

S1 Table The characterization of the 6 AU trajectories based on the LLCA class-specific probabilities of reporting each AU behavior from the LLCA. (DOCX)

S2 Table Selection of the best distribution with which to model the number of AUD criteria, based on likelihood-based methods (AIC, BIC (according to Schwarz, 1978), log-likelihood, and deviance). (DOCX)

S3 Table Pairwise comparisons of the number of AUD criteria across AU trajectories (K1-K6) at waves 1 and 3 under GLMM and GEE models. (DOCX)

S4 Table Pairwise comparisons of the number of AUD criteria across waves within each AU trajectory (K1–K6) under GLMM and GEE models. (DOCX)
