# Supplementary material for: Multiple trajectories of alcohol use and the development of alcohol use disorder: Do Swiss men mature-out of problematic alcohol use during emerging adulthood?
Source: PLoS One. 2020 Jan 27;15(1):e0220232. doi: 10.1371/journal.pone.0220232 (PMC6984690; doi:10.1371/journal.pone.0220232)
Supplement: S1 Supplementary Material — (DOCX) [file pone.0220232.s001.docx]

**S1 Supplementary material. Supplementary methods on the GMM and GEE.**

Count data are commonly modeled using either Poisson or negative binomial distributions. When data are over-dispersed, it is common to use a mixture of a Poisson or negative binomial distribution and a structural component that describes the variance. The structural component can model in relation to predictors of the presence of extra-zero (i.e., zero-inflated models) or the dispersion its-self (i.e., dispersion models). The best distribution with which to model the number of AUD criteria according to classes over time (i.e., the model including the interaction) was assessed by comparing models using (1) a Poisson or a negative binomial distribution and (2) a structural component to model either zero-inflation or dispersion depending on (3) time and classes in interaction or independently together, using the R software package ‘glmmTMB’ ([Brooks et al., 2017](#_ENREF_5)). The two parameterizations of the negative binomial distribution provided by glmmTMB were also assessed: for negative binomial 1, the variance increases linearly with the mean as σ^2^ = µ(1 + α), with α > 0; for negative binomial 2, the variance increases quadratically with the mean as σ^2^ = µ(1 + µ/θ), with θ > 0 ([Brooks et al., 2017](#_ENREF_5)).

To evaluate the robustness of the results, a generalized estimating equations model (i.e., GEE) was also used, with the R software package ‘geepack’ ([Højsgaard et al., 2005](#_ENREF_20)), to model the number of AUD criteria according to classes over time using *Poisson* as the distribution and *unstructured* as the correlation structure (which allows observations in a group to have different correlations).

Brooks, M. E., Kristensen, K., van Benthem, K. J., Magnusson, A., Berg, C. W., Nielsen, A., . . . Bolker, B. M. (2017). glmmTMB balances speed and flexibility among packages for zero-inflated generalized linear mixed modeling. *The R journal, 9*(2), 378-400.

Højsgaard, S., Halekoh, U., & Yan, J. (2005). The R Package geepack for Generalized Estimating Equations. *Journal of Statistical Software, Journal of Statistical Software* (2), 11. doi:10.18637/jss.v015.i02
