# Supplementary material for: Multiple trajectories of alcohol use and the development of alcohol use disorder: Do Swiss men mature-out of problematic alcohol use during emerging adulthood?
Source: PLoS One. 2020 Jan 27;15(1):e0220232. doi: 10.1371/journal.pone.0220232 (PMC6984690; doi:10.1371/journal.pone.0220232)
Supplement: S1 Table — (DOCX) [file pone.0220232.s002.docx]

**S1 Table. The characterization of the 6 AU trajectories based on the LLCA class-specific probabilities of reporting each AU behavior from the LLCA**

|  |  | AU trajectories | | | | | | | | | | |
| --- | --- | --- | --- | --- | --- | --- | --- | --- | --- | --- | --- | --- |
|  |  | K1 (N=467, 9.8%) | | |  | K2 (N=801, 16.9%) | | |  | K3 (N=576, 12.1%) | | |
| AU behavior | | W1 | W2 | W3 |  | W1 | W2 | W3 |  | W1 | W2 | W3 |
| **Drinking frequency on workweek days** | | | |  |  |  |  |  |  |  |  |  |
|  | *Never* | 0.96 | 0.95 | 0.84 |  | 0.56 | 0.44 | 0 |  | 0.51 | 0.49 | 0.99 |
|  | *Less than once per month* | 0.03 | 0.02 | 0.09 |  | 0.27 | 0.37 | 0.50 |  | 0.24 | 0.26 | 0 |
|  | *One day per month* | 0 | 0.01 | 0.03 |  | 0.08 | 0.07 | 0.18 |  | 0.07 | 0.08 | 0 |
|  | *Two or three days per month* | 0 | 0.01 | 0.02 |  | 0.06 | 0.08 | 0.16 |  | 0.07 | 0.07 | 0 |
|  | *One day per week* | 0 | 0 | 0.02 |  | 0.02 | 0.03 | 0.08 |  | 0.06 | 0.07 | 0 |
|  | *Two days per week* | 0 | 0 | 0 |  | 0 | 0.01 | 0.06 |  | 0.03 | 0.03 | 0 |
|  | *At least three days of the week* | 0 | 0 | 0 |  | 0 | 0 | 0.01 |  | 0.02 | 0.01 | 0 |
| **Drinking quantity on workweek days** | | |  |  |  |  |  |  |  |  |  |  |
|  | *None* | 0.96 | 0.95 | 0.84 |  | 0.56 | 0.44 | 0 |  | 0.50 | 0.49 | 0.98 |
|  | *One or two drinks per day* | 0.04 | 0.04 | 0.14 |  | 0.41 | 0.53 | 0.93 |  | 0.39 | 0.43 | 0.01 |
|  | *Three or four drinks per day* | 0 | 0 | 0.02 |  | 0.02 | 0.02 | 0.06 |  | 0.05 | 0.05 | 0 |
|  | *Five and more drinks per day* | 0 | 0 | 0.01 |  | 0 | 0.01 | 0.01 |  | 0.05 | 0.03 | 0 |
| **Drinking frequency on weekend days** | | | |  |  |  |  |  |  |  |  |  |
|  | *Never* | 0.73 | 0.74 | 0.61 |  | 0.07 | 0.06 | 0 |  | 0 | 0.03 | 0.19 |
|  | *Less than once per month* | 0.13 | 0.16 | 0.18 |  | 0.25 | 0.27 | 0.25 |  | 0.18 | 0.2 | 0.26 |
|  | *One day per month* | 0.04 | 0.04 | 0.08 |  | 0.18 | 0.21 | 0.18 |  | 0.13 | 0.14 | 0.16 |
|  | *Two or three days per month* | 0.04 | 0.02 | 0.05 |  | 0.23 | 0.26 | 0.26 |  | 0.20 | 0.20 | 0.15 |
|  | *One day per week* | 0.05 | 0.03 | 0.06 |  | 0.21 | 0.16 | 0.20 |  | 0.29 | 0.27 | 0.18 |
|  | *Two days per week* | 0.01 | 0.01 | 0.02 |  | 0.07 | 0.04 | 0.1 |  | 0.17 | 0.15 | 0.06 |
|  | *At least three days of the week* | 0 | 0 | 0.01 |  | 0 | 0 | 0.01 |  | 0.03 | 0.01 | 0 |
| **Drinking quantity on weekend days** | | | |  |  |  |  |  |  |  |  |  |
|  | *None* | 0.73 | 0.74 | 0.6 |  | 0.06 | 0.06 | 0 |  | 0 | 0.03 | 0.19 |
|  | *One or two drinks per day* | 0.20 | 0.22 | 0.28 |  | 0.52 | 0.57 | 0.54 |  | 0.32 | 0.33 | 0.35 |
|  | *Three or four drinks per day* | 0.04 | 0.02 | 0.07 |  | 0.26 | 0.29 | 0.35 |  | 0.29 | 0.26 | 0.26 |
|  | *Five or six drinks per day* | 0.03 | 0.01 | 0.03 |  | 0.11 | 0.07 | 0.08 |  | 0.18 | 0.23 | 0.14 |
|  | *Seven or eight drinks per day* | 0 | 0 | 0 |  | 0.03 | 0.01 | 0.02 |  | 0.07 | 0.08 | 0.04 |
|  | *Nine to eleven drinks per day* | 0 | 0 | 0.01 |  | 0.01 | 0.01 | 0.01 |  | 0.08 | 0.05 | 0.01 |
|  | *Twelve or more drinks per day* | 0 | 0 | 0.01 |  | 0.01 | 0 | 0.01 |  | 0.06 | 0.03 | 0.01 |
| **Frequency of HED** | |  |  |  |  |  |  |  |  |  |  |  |
|  | *Never* | 0.87 | 0.93 | 0.83 |  | 0.42 | 0.42 | 0.34 |  | 0.19 | 0.24 | 0.43 |
|  | *Less than once per month* | 0.11 | 0.06 | 0.13 |  | 0.5 | 0.56 | 0.6 |  | 0.50 | 0.51 | 0.50 |
|  | *Every month* | 0.01 | 0.01 | 0.02 |  | 0.06 | 0.02 | 0.06 |  | 0.18 | 0.16 | 0.05 |
|  | *Every week to every day* | 0 | 0 | 0.03 |  | 0.02 | 0 | 0.01 |  | 0.13 | 0.1 | 0.02 |

**S1 Table continued. The characterization of the 6 AU trajectories based on the LLCA class-specific probabilities of reporting each AU behavior from the LLCA**

|  |  | AU trajectories | | | | | | | | | | |
| --- | --- | --- | --- | --- | --- | --- | --- | --- | --- | --- | --- | --- |
|  |  | K4 (N=1327, 28.0%) | | |  | K5 (N=536, 11.3%) | | |  | K6 (N=1039, 21.9%) | | |
| AU behavior | | W1 | W2 | W3 |  | W1 | W2 | W3 |  | W1 | W2 | W3 |
| **Drinking frequency on workweek days** | | | |  |  |  |  |  |  |  |  |  |
|  | *Never* | 0 | 0.05 | 0 |  | 0.99 | 0.25 | 0.08 |  | 0.01 | 0.05 | 0.04 |
|  | *Less than once per month* | 0.34 | 0.22 | 0.23 |  | 0 | 0.24 | 0.26 |  | 0.15 | 0.08 | 0.08 |
|  | *One day per month* | 0.19 | 0.16 | 0.15 |  | 0 | 0.15 | 0.11 |  | 0.12 | 0.08 | 0.08 |
|  | *Two or three days per month* | 0.21 | 0.26 | 0.25 |  | 0 | 0.15 | 0.20 |  | 0.18 | 0.17 | 0.21 |
|  | *One day per week* | 0.18 | 0.21 | 0.22 |  | 0 | 0.13 | 0.21 |  | 0.24 | 0.31 | 0.25 |
|  | *Two days per week* | 0.06 | 0.08 | 0.11 |  | 0 | 0.06 | 0.10 |  | 0.18 | 0.21 | 0.22 |
|  | *At least three days of the week* | 0.03 | 0.03 | 0.03 |  | 0 | 0.01 | 0.04 |  | 0.12 | 0.10 | 0.12 |
| **Drinking quantity on workweek days** | | |  |  |  |  |  |  |  |  |  |  |
|  | *None* | 0 | 0.05 | 0 |  | 0.98 | 0.25 | 0.08 |  | 0 | 0.05 | 0.04 |
|  | *One or two drinks per day* | 0.90 | 0.80 | 0.87 |  | 0.01 | 0.57 | 0.66 |  | 0.66 | 0.55 | 0.55 |
|  | *Three or four drinks per day* | 0.08 | 0.12 | 0.11 |  | 0 | 0.12 | 0.18 |  | 0.23 | 0.26 | 0.29 |
|  | *Five and more drinks per day* | 0.02 | 0.03 | 0.02 |  | 0.01 | 0.06 | 0.09 |  | 0.11 | 0.14 | 0.13 |
| **Drinking frequency on weekend days** | | | |  |  |  |  |  |  |  |  |  |
|  | *Never* | 0 | 0 | 0 |  | 0.05 | 0 | 0 |  | 0 | 0 | 0 |
|  | *Less than once per month* | 0.02 | 0.02 | 0.04 |  | 0.04 | 0.02 | 0.06 |  | 0 | 0 | 0.01 |
|  | *One day per month* | 0.07 | 0.06 | 0.08 |  | 0.06 | 0.05 | 0.07 |  | 0.01 | 0.01 | 0.02 |
|  | *Two or three days per month* | 0.20 | 0.16 | 0.21 |  | 0.17 | 0.14 | 0.15 |  | 0.02 | 0.02 | 0.08 |
|  | *One day per week* | 0.34 | 0.39 | 0.34 |  | 0.35 | 0.38 | 0.32 |  | 0.16 | 0.19 | 0.25 |
|  | *Two days per week* | 0.34 | 0.33 | 0.29 |  | 0.32 | 0.36 | 0.35 |  | 0.65 | 0.62 | 0.49 |
|  | *At least three days of the week* | 0.03 | 0.04 | 0.04 |  | 0.01 | 0.05 | 0.06 |  | 0.16 | 0.17 | 0.15 |
| **Drinking quantity on weekend days** | | |  |  |  |  |  |  |  |  |  |  |
|  | *None* | 0 | 0 | 0 |  | 0.04 | 0 | 0 |  | 0 | 0 | 0 |
|  | *One or two drinks per day* | 0.15 | 0.17 | 0.26 |  | 0.10 | 0.08 | 0.09 |  | 0.01 | 0.01 | 0.03 |
|  | *Three or four drinks per day* | 0.42 | 0.44 | 0.44 |  | 0.28 | 0.28 | 0.29 |  | 0.08 | 0.09 | 0.19 |
|  | *Five or six drinks per day* | 0.28 | 0.26 | 0.22 |  | 0.27 | 0.32 | 0.32 |  | 0.27 | 0.27 | 0.26 |
|  | *Seven or eight drinks per day* | 0.08 | 0.09 | 0.05 |  | 0.14 | 0.15 | 0.17 |  | 0.23 | 0.28 | 0.22 |
|  | *Nine to eleven drinks per day* | 0.04 | 0.03 | 0.02 |  | 0.09 | 0.10 | 0.08 |  | 0.20 | 0.18 | 0.16 |
|  | *Twelve or more drinks per day* | 0.02 | 0.01 | 0 |  | 0.07 | 0.07 | 0.05 |  | 0.20 | 0.18 | 0.13 |
| **Frequency of HED** | |  |  |  |  |  |  |  |  |  |  |  |
|  | *Never* | 0.04 | 0.03 | 0.06 |  | 0.11 | 0.01 | 0.03 |  | 0 | 0 | 0 |
|  | *Less than once per month* | 0.47 | 0.54 | 0.58 |  | 0.37 | 0.35 | 0.36 |  | 0.04 | 0.04 | 0.15 |
|  | *Every month* | 0.39 | 0.36 | 0.30 |  | 0.31 | 0.39 | 0.39 |  | 0.29 | 0.30 | 0.38 |
|  | *Every week to every day* | 0.09 | 0.07 | 0.06 |  | 0.22 | 0.25 | 0.22 |  | 0.67 | 0.65 | 0.47 |
