# Supplementary material for: Multiple trajectories of alcohol use and the development of alcohol use disorder: Do Swiss men mature-out of problematic alcohol use during emerging adulthood?
Source: PLoS One. 2020 Jan 27;15(1):e0220232. doi: 10.1371/journal.pone.0220232 (PMC6984690; doi:10.1371/journal.pone.0220232)
Supplement: S2 Table — (DOCX) [file pone.0220232.s003.docx]

**S2 Table. Selection of the best distribution with which to model the number of AUD criteria, based on likelihood-based methods (AIC, BIC (according to** [**Schwarz, 1978**](#_ENREF_42)**), log-likelihood, and deviance)**

| Family | Structural component | AIC | BIC | logLik | deviance | df |
| --- | --- | --- | --- | --- | --- | --- |
| Poisson | - | 38338 | 38482 | -19150 | 38300 | 14138 |
| negative binomial 1 | - | 38130 | 38282 | -19045 | 38090 | 14137 |
| negative binomial 2 | - | 38311 | 38462 | -19135 | 38271 | 14137 |
| Poisson | zero-inflation by time | 38301 | 38468 | -19129 | 38257 | 14135 |
| negative binomial 1 | zero-inflation by time | 38136 | 38310 | -19045 | 38090 | 14134 |
| negative binomial 2 | zero-inflation by time | 38301 | 38475 | -19128 | 38255 | 14134 |
| Poisson | dispersion by time | cannot be calculated | | |  |  |
| negative binomial 1 | dispersion by time | 38101 | 38267 | -19028 | 38057 | 14135 |
| negative binomial 2 | dispersion by time | 38281 | 38448 | -19119 | 38237 | 14135 |
| Poisson | zero-inflation by classes | 37991 | 38180 | -18971 | 37941 | 14132 |
| negative binomial 1 | zero-inflation by classes | 37993 | 38189 | -18970 | 37941 | 14131 |
| negative binomial 2 | zero-inflation by classes | 37993 | 38190 | -18971 | 37941 | 14131 |
| Poisson | dispersion by classes | cannot be calculated | | |  |  |
| negative binomial 1 | dispersion by classes | 37867 | 38056 | -18909 | 37817 | 14132 |
| negative binomial 2 | dispersion by classes | 37924 | 38113 | -18937 | 37874 | 14132 |
| Poisson | zero-inflation by classes and time | 37980.8 | 38184.9 | -18963.4 | 37926.8 | 14130 |
| negative binomial 1 | zero-inflation by classes and time | 37982.7 | 38194.4 | -18963.4 | 37926.7 | 14129 |
| negative binomial 2 | zero-inflation by classes and time | 37982.8 | 38194.4 | -18963.4 | 37926.8 | 14129 |
| Poisson | dispersion by classes and time | cannot be calculated | | |  |  |
| negative binomial 1 | dispersion by classes and time | ***37850*** | ***38054*** | -18898 | 37796 | 14130 |
| negative binomial 2 | dispersion by classes and time | 37912 | 38117 | -18929 | 37858 | 14130 |
| negative binomial 1 | dispersion by classes per time | 37851 | 38131 | ***-18889*** | ***37777*** | 14120 |

AIC: Akaike information criterion; BIC: Bayesian information criterion; logLik: log-likelihood; df: degree of freedom. Because, in a Poisson distribution, variance equals mean, a dispersion model cannot be calculated.

The model including the predictors in the structural dispersion component always outcompeted the model including the predictors in the structural zero-inflated component. Moreover, the negative binomial 1 (variance increasing linearly with the mean) generally outcompeted the Poisson distribution and the negative binomial 2 (variance increasing quadratically with the mean). Including the interaction between time and classes improved the model’s fit (log-likelihood (logLik) and deviance) but used 10 additional parameters and was therefore penalized based on information criteria (AIC and BIC) in comparison to the additive model (classes and time).
