# Supplementary material for: Multiple trajectories of alcohol use and the development of alcohol use disorder: Do Swiss men mature-out of problematic alcohol use during emerging adulthood?
Source: PLoS One. 2020 Jan 27;15(1):e0220232. doi: 10.1371/journal.pone.0220232 (PMC6984690; doi:10.1371/journal.pone.0220232)
Supplement: S3 Table — (DOCX) [file pone.0220232.s004.docx]

**S3 Table. Pairwise comparisons of the number of AUD criteria across AU trajectories (K1-K6) at waves 1 and 3 under GLMM and GEE models.**

|  | Pairwise comp. | | GLMM | | | |  | GEE | | | |
| --- | --- | --- | --- | --- | --- | --- | --- | --- | --- | --- | --- |
|  | P1 | P2 | Estimate | SE | z value | P-value |  | Estimate | SE | Wald value | P-value |
| *Wave 1* | |  |  |  |  |  |  |  |  |  |  |
|  | K1 | K6 | 3.323 | 0.250 | 13.27 | 0.000 |  | 3.305 | 0.279 | 140.69 | 0.000 |
|  | K1 | K4 | 2.613 | 0.250 | 10.44 | 0.000 |  | 2.619 | 0.279 | 87.88 | 0.000 |
|  | K1 | K5 | 2.513 | 0.254 | 9.90 | 0.000 |  | 2.527 | 0.284 | 79.29 | 0.000 |
|  | K1 | K3 | 2.337 | 0.255 | 9.16 | 0.000 |  | 2.332 | 0.287 | 65.94 | 0.000 |
|  | K1 | K2 | ***1.711*** | ***0.255*** | ***6.70*** | ***0.000*** |  | ***1.712*** | ***0.286*** | ***35.76*** | ***0.000*** |
|  | K2 | K6 | 1.613 | 0.075 | 21.48 | 0.000 |  | 1.593 | 0.075 | 453.60 | 0.000 |
|  | K2 | K4 | 0.903 | 0.076 | 11.82 | 0.000 |  | 0.907 | 0.078 | 136.81 | 0.000 |
|  | K2 | K5 | 0.802 | 0.089 | 9.04 | 0.000 |  | 0.815 | 0.092 | 78.21 | 0.000 |
|  | K2 | K3 | ***0.626*** | ***0.096*** | ***6.51*** | ***0.000*** |  | ***0.620*** | ***0.102*** | ***36.91*** | ***0.000*** |
|  | K3 | K6 | 0.987 | 0.074 | 13.34 | 0.000 |  | 0.974 | 0.078 | 155.95 | 0.000 |
|  | K3 | K4 | 0.277 | 0.075 | 3.68 | 0.000 |  | 0.288 | 0.081 | 12.73 | 0.000 |
|  | K3 | K5 | ***0.176*** | ***0.088*** | ***2.00*** | ***0.046*** |  | ***0.196*** | ***0.095*** | ***4.26*** | ***0.039*** |
|  | K5 | K6 | 0.810 | 0.062 | 13.01 | 0.000 |  | 0.778 | 0.065 | 145.06 | 0.000 |
|  | K5 | K4 | ***0.100*** | ***0.064*** | ***1.57*** | ***0.116*** |  | ***0.092*** | ***0.068*** | ***1.85*** | ***0.174*** |
|  | K4 | K6 | ***0.710*** | ***0.041*** | ***17.20*** | ***0.000*** |  | ***0.686*** | ***0.041*** | ***277.70*** | ***0.000*** |
| *Wave 3* | |  |  |  |  |  |  |  |  |  |  |
|  | K1 | K6 | 2.844 | 0.171 | 16.66 | 0.000 |  | 2.891 | 0.168 | 297.12 | 0.000 |
|  | K1 | K5 | 2.374 | 0.176 | 13.50 | 0.000 |  | 2.443 | 0.172 | 201.96 | 0.000 |
|  | K1 | K4 | 2.139 | 0.172 | 12.46 | 0.000 |  | 2.205 | 0.168 | 171.38 | 0.000 |
|  | K1 | K2 | 1.518 | 0.177 | 8.55 | 0.000 |  | 1.607 | 0.175 | 83.93 | 0.000 |
|  | K1 | K3 | ***1.310*** | ***0.184*** | ***7.14*** | ***0.000*** |  | ***1.401*** | ***0.185*** | ***57.46*** | ***0.000*** |
|  | K3 | K6 | 1.534 | 0.080 | 19.27 | 0.000 |  | 1.490 | 0.086 | 301.58 | 0.000 |
|  | K3 | K5 | 1.064 | 0.088 | 12.13 | 0.000 |  | 1.042 | 0.094 | 123.55 | 0.000 |
|  | K3 | K4 | 0.829 | 0.081 | 10.28 | 0.000 |  | 0.803 | 0.087 | 85.00 | 0.000 |
|  | K3 | K2 | ***0.208*** | ***0.092*** | ***2.26*** | ***0.024*** |  | ***0.206*** | ***0.100*** | ***4.23*** | ***0.040*** |
|  | K2 | K6 | 1.326 | 0.061 | 21.61 | 0.000 |  | 1.284 | 0.063 | 417.27 | 0.000 |
|  | K2 | K5 | 0.857 | 0.072 | 11.86 | 0.000 |  | 0.836 | 0.073 | 129.98 | 0.000 |
|  | K2 | K4 | ***0.621*** | ***0.063*** | ***9.87*** | ***0.000*** |  | ***0.598*** | ***0.065*** | ***85.32*** | ***0.000*** |
|  | K4 | K6 | 0.705 | 0.041 | 17.09 | 0.000 |  | 0.686 | 0.039 | 305.09 | 0.000 |
|  | K4 | K5 | ***0.235*** | ***0.056*** | ***4.21*** | ***0.000*** |  | ***0.238*** | ***0.055*** | ***19.12*** | ***0.000*** |
|  | K5 | K6 | ***0.469*** | ***0.054*** | ***8.65*** | ***0.000*** |  | ***0.448*** | ***0.052*** | ***73.35*** | ***0.000*** |

Estimates were extracted from the linear models and were not back-transformed according to the link function. Pairwise comparisons within waves were ordered from larger to smaller differences by class, from K1 to K6. Bold italics highlight the smallest differences by class, from K1 to K6. The order of the waves was not included in the GEE. The number of AUD criteria varied among the six AU trajectories, both in terms of initial level and temporal variation (interaction classes x time: GLMM: LRT *χ*^2^_10_ = 104.00, *P* < 0.001; GEE: MWT *χ*^2^_10_ = 81.25, *P* < 0.001).
