# Supplementary material for: Multiple trajectories of alcohol use and the development of alcohol use disorder: Do Swiss men mature-out of problematic alcohol use during emerging adulthood?
Source: PLoS One. 2020 Jan 27;15(1):e0220232. doi: 10.1371/journal.pone.0220232 (PMC6984690; doi:10.1371/journal.pone.0220232)
Supplement: S4 Table — (DOCX) [file pone.0220232.s005.docx]

**S4 Table. Pairwise comparisons of the number of AUD criteria across waves within each AU trajectory (K1–K6) under GLMM and GEE models.**

|  | Pairwise comp. | | GLMM | | | |  | GEE | | | |
| --- | --- | --- | --- | --- | --- | --- | --- | --- | --- | --- | --- |
| Class | P1 | P2 | Estimate | SE | z value | *P*-value |  | Estimate | SE | Wald value | *P*-value |
| K1 | W1 | W2 | -0.107 | 0.429 | -0.249 | 0.803 |  | -0.012 | 0.374 | 0.001 | 0.975 |
| K1 | W2 | W3 | ***0.551*** | ***0.271*** | ***2.032*** | ***0.042*** |  | 0.394 | 0.313 | 1.587 | 0.208 |
| K1 | W1 | W3 | 0.448 | 0.297 | 1.510 | 0.131 |  | 0.382 | 0.315 | 1.470 | 0.225 |
| K2 | W1 | W2 | -0.117 | 0.091 | -1.285 | 0.199 |  | -0.107 | 0.078 | 1.862 | 0.172 |
| K2 | W2 | W3 | ***0.372*** | ***0.080*** | ***4.664*** | ***0.000*** |  | ***0.384*** | ***0.079*** | ***23.432*** | ***0.000*** |
| K2 | W1 | W3 | ***0.255*** | ***0.082*** | ***3.096*** | ***0.002*** |  | ***0.277*** | ***0.083*** | ***11.030*** | ***0.001*** |
| K3 | W1 | W2 | ***-0.182*** | ***0.087*** | ***-2.082*** | ***0.037*** |  | ***-0.170*** | ***0.084*** | ***4.113*** | ***0.043*** |
| K3 | W2 | W3 | ***-0.397*** | ***0.092*** | ***-4.325*** | ***0.000*** |  | ***-0.378*** | ***0.100*** | ***14.212*** | ***0.000*** |
| K3 | W1 | W3 | ***-0.579*** | ***0.094*** | ***-6.151*** | ***0.000*** |  | ***-0.548*** | ***0.108*** | ***25.807*** | ***0.000*** |
| K4 | W1 | W2 | -0.067 | 0.037 | -1.831 | 0.067 |  | ***-0.071*** | ***0.036*** | ***3.898*** | ***0.048*** |
| K4 | W2 | W3 | 0.041 | 0.036 | 1.129 | 0.259 |  | 0.039 | 0.035 | 1.225 | 0.268 |
| K4 | W1 | W3 | -0.026 | 0.036 | -0.717 | 0.473 |  | -0.032 | 0.037 | 0.761 | 0.383 |
| K5 | W1 | W2 | ***0.250*** | ***0.063*** | ***3.946*** | ***0.000*** |  | ***0.249*** | ***0.062*** | ***15.856*** | ***0.000*** |
| K5 | W2 | W3 | 0.059 | 0.056 | 1.055 | 0.291 |  | 0.049 | 0.054 | 0.850 | 0.356 |
| K5 | W1 | W3 | ***0.309*** | ***0.062*** | ***5.002*** | ***0.000*** |  | ***0.298*** | ***0.061*** | ***23.612*** | ***0.000*** |
| K6 | W1 | W2 | -0.011 | 0.028 | -0.405 | 0.685 |  | -0.012 | 0.026 | 0.214 | 0.643 |
| K6 | W2 | W3 | -0.021 | 0.028 | -0.733 | 0.464 |  | -0.020 | 0.026 | 0.579 | 0.447 |
| K6 | W1 | W3 | -0.032 | 0.028 | -1.138 | 0.255 |  | -0.032 | 0.029 | 1.252 | 0.263 |

Estimates were extracted from the linear models and were not back-transformed according to the link function. Bold italics highlight significant pairwise comparisons at *P* < 0.05. The number of AUD criteria varied among the six AU trajectories, both in terms of initial level and temporal variation (interaction classes x time: GLMM: LRT *χ*^2^_10_ = 104.00, *P* < 0.001; GEE: MWT *χ*^2^_10_ = 81.25, *P* < 0.001).
